# Autonomic stress reactivity and craving in individuals with problematic Internet use

**DOI:** 10.1371/journal.pone.0190951

**Published:** 2018-01-16

**Authors:** Tania Moretta, Giulia Buodo

**Affiliations:** Department of General Psychology, University of Padova, Padova, Italy; Sichuan University West China Hospital, CHINA

## Abstract

The link between autonomic stress reactivity and subjective urge/craving has been less systematically examined in behavioral addictions (i.e. problematic Internet use) than in substance use disorders. The present study investigated whether problematic Internet users (PU) show enhanced autonomic stress reactivity than non-PU, indexed by lower Heart Rate Variability (HRV) and higher Skin Conductance Level (SCL) reactivity during the Trier Social Stress Test (TSST), whether greater reactivity is related to stronger Internet craving, and whether problematic Internet usage is associated with some dysfunctional psychological features. Based on their Internet Addiction Test scores, participants were divided into PU (N = 24) and non-PU (N = 21). Their heart rate and skin conductance were continuously recorded during baseline, social stressors, and recovery. Craving for Internet usage were collected using a Likert scale before and after the TSST. The SDNN, an overall measure of HRV, was significantly lower in PU than non-PU during baseline, but not during and after stressful task. Furthermore, only among PU a significant negative correlation emerged between SDNN during recovery and craving ratings after the test. No group differences emerged for SCL. Lastly, PU endorsed more mood, obsessive-compulsive, and alcohol-related problems. Our findings suggest that problems in controlling one’s use of the Internet may be related to reduced autonomic balance at rest. Moreover, our results provide new insights into the characterization of craving in PIU, indicating the existence of a relationship between craving for Internet usage and reduced autonomic flexibility.

## Introduction

In the last decade, the Internet has become part of our daily life, changing the way we work and communicate. Despite its advantages, many people spend more time than necessary on the Internet and sometimes a psychopathological condition may result. Despite the growing number of studies in this context, researchers are still not yet in agreement on the conceptualization of Internet-related psychological problems, and a specific diagnosis is not included in any established diagnostic system of mental disorders [1–3]. Psychological problems regarding Internet use were first described as Internet Addiction Disorder, defined as an impulse-control disorder that does not involve an intoxicant [4]. Since then, different labels have been used in the scientific literature to capture Internet-related problems, including Internet addiction, compulsive Internet use, and Problematic Internet Use (PIU) [5]. PIU refers to “Internet use that creates psychological, social, school, and/or work difficulties in a person’s life” [6,7]. The dominant perspective conceptualizes PIU as an addictive behavior [4,8,9], thus suggesting that PIU, similarly to substance use disorders (SUDs) and behavioral addictions (BA; e.g., pathological gambling), is characterized by the persistence of a specific behavior (Internet use) despite its adverse effects [10].

A number of different psychometric tools have been developed to assess PIU, among which the Assessment of Internet and Computer Game Addiction Scale (AICA-S, [11], the Compulsive Internet Use Scale (CIUS) [12], and the Young’s Internet Addiction Test (IAT) [4]. The IAT has been used in the majority of studies on PIU, although comparison among studies is made difficult by the fact that different cut-off scores have been used to classify participants [13,14].

Several psychopathological disorders have been found to be often comorbid with PIU. It has been recently reported that individuals with PIU are more likely to have psychiatric disorders or symptoms including SUDs, mood disorders, anxiety disorders, somatoform disorders, pathological gambling, Attention-Deficit Hyperactivity Disorder symptoms, sleep disturbances and suicide ideation [15], obsessive-compulsive symptoms [16], and alexithymia [17]. Such findings suggest that it is important to carefully assess the presence of such conditions in individuals with PIU.

In addition to assessing and managing comorbidities, identifying the factors that precipitate and maintain PIU is of fundamental importance. Studies on SUDs have shown that craving is a key factor in the maintenance of addictive behaviors [18]. Craving is defined as a subjective motivational state involving an intense urge to engage in a specific behavior, and is thought to reflect a conditioned response resulting from repeated pairing of stimuli with reward [19]. A recent study showed that, among individuals with PIU, exposure to Internet-related words was followed by an increase in craving ratings, suggesting that PIU may share similar underlying mechanisms with other addiction disorders [20].

Research has increasingly recognized the importance of understanding the relationship between PIU and stress, including the role of potential mediators and moderators [2]. Stress occurs when an individual perceives that environmental demands exceed his or her adaptive capacity [21]. Studies on addictions suggest that acute and/or chronic stress can determine the attribution of additional salience to reward-related stimuli [22,23], favoring the formation of conditioned responses without an appraisal of response consequences, i.e., habits [24]. Habits are believed to be at the basis of craving [25–27]. Specifically, Schwabe and colleagues [26–28] proposed that acute or chronic stress cause the release of several hormones, including glucocorticoids, noradrenaline and adrenaline, that facilitate striatum-dependent memory processes by favoring dorsolateral striatum-based habits.

The link between stress reactivity and craving has been less systematically examined in behavioral addictions than in SUDs. However, it can be hypothesized that Internet-related habits are potentiated and reinstated by stress, leading to craving symptoms and PIU establishment. In other words, an exaggerated reactivity to stressors may lead to an excessive engagement of habit processes in instrumental action, thus promoting conditioned habitual responses to Internet-related stimuli at the basis of compulsive Internet use [29,30].

Few studies have investigated the relationship between stress reactivity and PIU using self-report measures. Specifically, it has been shown that stressful life events were positively correlated with Internet addiction [31]. Furthermore, perceived stress has been found to be one of the predictors of PIU for sexual purposes [32], and PIU appears to be associated with stress, depression and anxiety symptoms [33]. No study to our knowledge has yet investigated stress reactivity in individuals with PIU using psychophysiological indices in addition to self-report instruments.

In the assessment of psychophysiological indices of the stress response, both the magnitude of response and the capacity to recover (i.e., the degree to which a psychophysiological response returns to pre-stress levels following a stressor) have been commonly considered as relevant parameters. Classical laboratory stress tasks used to investigate psychophysiological stress responses include public speaking and mental arithmetic, and the most commonly assessed indices include autonomic measures such as heart rate and heart rate variability (HRV) and skin conductance (SC).

HRV consists in the variations over time of the period between consecutive heartbeats (RR intervals). Such variations represent a fine tuning of the beat-to-beat control mechanisms by vagal and sympathetic activity directed to the sinus node of the heart [34]. High HRV reflects the autonomic nervous system (ANS) ability to adapt to changing circumstances, and it seems to be associated with goal-based control of emotions, context-appropriate responses and recovery after stressor [35]. In contrast, low HRV reflects the ANS inability to adapt to stressful events and is associated with delayed recovery from psychological stress [36].

The analysis of the spectral components of HRV allows to understand the modulatory effects of neural mechanisms on the sinus node. In particular, the high frequency (HF; .15-.4 Hz) component is mainly determined by efferent vagal activity, whereas the low frequency (LF; .04-.15 Hz) component is considered by some as a marker of sympathetic modulation and by others as a parameter that includes both sympathetic and vagal influences [34]. In response to stressors, an increase in sympathetic cardiac control, a decrease in parasympathetic control, or both, are often observed, as reflected by increase in LF, a decrease in HF power, and/or an increase in the LF/HF ratio [37].

Skin conductance (SC) is a non-invasive measure of the variations in electrical conductance of the skin depending on the changes in the levels of sweat in the ducts [38]. SC reflects only the activity of the sympathetic component of the ANS, due to the absence of parasympathetic innervation on eccrine sweat glands. SC has been largely measured to assess sympathetic activation during challenging situations [39,40].

The goals of the present study were to investigate (i) whether individuals with PIU show enhanced autonomic reactivity to a standardized psychosocial stress task; (ii) whether greater autonomic reactivity is related to higher craving ratings; and (iii) whether the presence of PIU is associated with high levels of anxiety, depression, impulsivity, alexithymia, obsessive-compulsive symptoms and more frequent use of alcohol and cannabis.

We hypothesized that PIU individuals would be characterized by lower HRV and higher SC level during the stress task as compared with individuals without PIU. Furthermore, we expected to observe an increase of craving ratings after the stress task in individuals with, but not in individuals without, PIU. Lastly, we expected that individuals with PIU would show higher scores on self-reports of anxiety, depression, impulsivity, alexithymia, obsessive-compulsive symptoms and use of alcohol and cannabis than individuals without PIU.

## Materials and methods

### Participants

Students of the University of Padua, Italy, were contacted informally at university facilities and asked to fill in an anonymous online version of the IAT [4; Italian version by 41]. The IAT is a 20-item questionnaire that measures six factors at the basis of PIU, i.e., compromised social and individual quality of life, careers, and time control, and excitatory/compensatory usage of the Internet. Based on Italian cut-off scores, Internet usage was defined as non problematic (scores 20–50), occasional or frequent problematic (scores 50–80), and severe problematic (scores 80–100) [42].

188 students filled in the online questionnaire. Twenty-four students who qualified as problematic Internet users (PU; 15 females; mean age = 23.04 ± 3.57; mean IAT score = 58 ± 7.2, range = 49–71), and 21 who qualified as non-problematic Internet users (non-PU; 17 females, mean age = 23.29 ± 2.87; mean IAT score = 31 ± 4.6, range = 23–39) accepted to participate in the study. No age, gender, sleep hours, and cigarettes consumption differences between groups were found.

Approval for the study was obtained from the Ethical Committee of Psychological Research, Area 17, University of Padova (prot. N. 1887).

### Self-report measures

The Italian version of the Alcohol Use Disorders Identification Test (AUDIT) [43] was used to assess the frequency and quantity of alcohol consumption [44]. Score ranges from 0 to 40, with higher scores indicating more problematic alcohol use.

The Italian version of the Cannabis Abuse Screening Test (CAST) [45] was administered to assess cannabis use with reference to the past 12 months. Score ranges from 0 to 24. Cut-off score for problematic cannabis use is 7.

The Italian version of the Depression Anxiety Stress Scales-21 (DASS-21) [46] was administered to assess general distress through three separate subscales (i.e., anxiety, depression, and stress).

The Italian version of the Barratt Impulsiveness Scale (BIS-11) [47] was administered to assess impulsivity. The higher the total score (range = 30–120), the higher the impulsiveness level.

The Italian version of the short UPPS-P Impulsive Behaviour Scale [48] was administered to assess five components of impulsivity: positive urgency, negative urgency, lack of perseverance, lack of premeditation, and sensation seeking.

The Italian version of the Obsessive-Compulsive Inventory-Revised (OCI-R) [49] was used to measure obsessive-compulsive symptoms.

The Italian version of the Toronto Alexithymia Scale (TAS-20) [50] was used to assess alexithymia symptoms.

See data in S2 File.

### Craving measure

To assess craving for Internet use, participants were asked to respond to a single question (“How much would you like to use the Internet now?”) using a Likert scale (range 1–5; 1 = not at all, 5 = very much). See data in S2 File.

### Stress task

A modified version of the Trier Social Stress Test (TSST) [51] was employed. Participants were first invited to remain quiet (Phase 1; 3 minute-baseline). Then, they were asked to prepare an oral speech about their personal traits qualifying them for their “dream” job position (Phase 2; 3 minutes). In the following phase, they were asked to speak in front of a video camera (Phase 3; 5 minutes). Participants were informed that video camera was connected to a monitor in another room, where an evaluation commission would judge their performance. Then the experimenter invited participants to rest again for six minutes (Phase 4, 3-minute recovery; and Phase 5, 3-minute baseline). In the following phase (Phase 6, 5 minutes), participants were asked to start counting backwards in steps of 13, starting at 2011. Upon each error, the experimenter asked them to start over. Lastly, participants were invited to rest again for three minutes (Phase 7).

### Autonomic measures

The electrocardiogram (ECG) and skin conductance (SC) were recorded continuously using a ProComp Infiniti system (Thought Technology; Montreal, Canada). To record the ECG, three disposable Ag/AgCl electrodes were placed on the participant's chest in a modified lead II configuration. The ECG signal was sampled at 256 Hz, band-pass filtered (1–100 Hz), and amplified. A digital trigger detecting R-waves was applied to the ECG signal to obtain inter-beat intervals (IBIs). All ECG data were visually examined and artifacts were corrected. Time domain and frequency domain indices of HRV were compute by Kubios HRV Analysis Software 2.0 (The Biomedical Signal Analysis Group, Department of Applied Physics, University of Kuopio, Finland). Fourier analysis was used to calculate frequency domain indices, i.e., low frequency power (LF: 0.04 to 0.15 Hz) in ms^2^, considered as an index of both ANS branches activity; High frequency power (HF: 0.15 to 0.40 Hz) in ms^2^, a HRV index of cardiac parasympathetic tone. As time domain indices, the standard deviation of all normal-to-normal intervals (SDNN) was calculated as an index of the total HRV, and the root mean square of successive difference of N-to-N intervals (rMSSD), expressed in ms, was calculated as an index of vagal control on the heart [34].

Skin Conductance Level (SCL) was recorded by two Ag/AgCl electrodes fixed to the medial phalanx surface of the first and middle finger of the nondominant hand Sampling rate was 256 Hz.

See data in S2 File.

### Procedure

After participants provided a written informed consent, they were asked to rate their Internet craving using the Likert scale. Then, ECG and SC sensors were placed and participants were given instructions about the task. After completion of the task, participants were asked again to rate their Internet craving on the Likert scale and sensors were removed. After the experimental session, the participants were asked to fill-in the questionnaires. The entire procedure took about 40 min.

### Statistical analysis

All statistical analyses were conducted on the mean values of SDNN, rMSSD, HF, LF, HF/LF ratio, and SCL calculated over the 3-min interval of Phases 1, 2, 4, 5 and 7, and the central 3 minutes in the 5-min Phases 3 and 6.

All analyses were performed using R software [52]. Specifically, Pearson’s r (R package: Hmisc) [53] was calculated to assess the strengths of correlations between self-report measures in both PU and non-PU.

To test autonomic reactivity during the TSST we estimated fifty mixed-models (Formulae A in S1 File by R package: lme4) [54] and the best-fitting model was selected using the AIC criteria [55,56], i.e., the model with the smallest AIC and the highest AIC weight is considered as the most appropriate model for reproducing the observed data. Mixed-effects models are considered as a powerful procedure for repeated-measures designs in psychophysiology [57]. Considering autonomic indices as dependent variables, the mixed-models were defined by starting from a simple model with individuals (i) random intercept only (Model 0; see Formulae A in S1 File: *Y*_*ij*_ = *b*_0_ + *v*_*i*_ + *e*_*ij*_, where *Y*_*ij*_ was the response for j^th^ measurement of i^th^ individual; *b*_0_ was the fixed intercept; *v*_*i*_ was the random intercept fot the i^th^ individual and *e*_*ij*_ was a Gaussian error term) and adding one fixed predictor to each subsequent model. Fixed predictors included Group (PU and non-PU), Phase (TSST phases), their interaction, and self-report measures that had been observed to be significantly reciprocally correlated in each Group. Hypothesized group differences in stress reactivity were fitted adding Group, Phase, and their interaction as fixed factors (Model 46; see Formulae A in S1 File) to Model 0. The maximum likelihood method was employed to analyze the contribution of parameters within the selected model (the modeling approach utilized data of all participants, except for SCL, for which one participant was excluded due to marked deviation from all other observations in the sample).

To assess whether Group (PU and non-PU), Time (before and after the TSST) and their interaction predict craving ratings (R package: MASS) [58] we estimated five nested ordinal logistic models and the AIC criteria were employed to select the model that more appropriately described our data [55,56].

Linear model analysis considering Group (PU and non-PU) as predictor was performed to compare scores on self-reports between groups. Bayes factor analysis was run to quantify the predictive success of linear models with Group predictor relative to an intercept-only model (R package: BayesFactor) [59].

## Results

### Autonomic measures

Descriptive statistics of autonomic indices are reported in Table 1.

**Table 1:** Descriptive statistics of autonomic measures.

		PU				non-PU			
TSST phases	Index	mean	sd	median	range	mean	sd	median	Range
Phase1	SDNN	66.23	28.38	63.06	96	80.21	32.6	78.08	124.43
	rMSSD	39.95	18.69	38.24	82.86	45.43	28.43	33.98	127.77
	LF (ms^2^)	915.43	455.43	901.08	1976.5	1317.8	1140.2	1023	4829.7
	HF (ms^2^)	932.06	957.74	783.96	4552.27	1296.5	2365.4	733.59	11135
	SCL	1.93	2.21	1.43	11.15	1.7	1.48	1.42	6.61
Phase 2	SDNN	58.55	16.63	54.19	56.19	56.24	20.59	50.75	81.52
	rMSSD	40.44	17.73	38.2	72.39	42.28	23.61	35.42	104.71
	LF (ms^2^)	1058.05	847.64	694.28	3243.61	990.88	978.25	639.55	3730.8
	HF (ms^2^)	1025.46	1312.99	547.31	4473.59	905.71	949.02	570.17	3745.6
	SCL	3.54	3.28	2.54	15.57	3.06	2.33	2.3	8.52
Phase3	SDNN	54.61	18.83	53.82	81.13	53.06	20.63	50.87	68.88
	rMSSD	32.41	13.98	31.5795	65.15	35.63	18.46	33.32	62.1
	LF (ms^2^)	1148	856.9	1098.42	2864.45	1381.6	1614	523.71	5144.3
	HF (ms^2^)	616.1	508.21	604.81	2456.82	685.93	914.53	466.46	4002.1
	SCL	4.53	4.17	2.96	20.51	3.97	3.34	2.84	11.46
Phase4	SDNN	57.56	16.79	55.48	58.72	64.36	22.91	59.26	79.27
	rMSSD	37.35	20.78	30.04	80.83	41.33	27.3	33.41	116.83
	LF (ms^2^)	1365.97	1058.1	976.86	4271.03	1551.4	1035.4	1203.7	4106.5
	HF (ms^2^)	716.55	809.94	449.58	3326.49	1046.1	1694.9	426.96	7663.1
	SCL	4.22	4.58	2.91	22.45	3.61	3.28	2.24	10.12
Phase5	SDNN	60.12	22.02	54.86	94.8	61.95	17.08	63.11	69.26
	rMSSD	47.51	30.69	39.5	143.84	49.72	22.12	46.64	101.12
	LF (ms^2^)	1253.989	1111.24	770.8	4259.27	1173.6	1351.2	697.82	6004.9
	HF (ms^2^)	1178.72	1400.36	750.63	6240.42	1367	1550.2	1010.7	6759.4
	SCL	3.92	4.67	2.74	22.8	3.34	3.40	1.75	11.52
Phase 6	SDNN	58.59	14.6	56.97	57.73	57.76	19.64	54.06	70.4
	rMSSD	39.84	15.76	41.39	64.21	43.25	18.06	36.77	59.81
	LF (ms^2^)	1561.15	1087.48	1306.3	5123.18	1428.9	1693.9	949.61	7705.8
	HF (ms^2^)	1022.81	892.13	672.71	3046.49	963.51	840.33	778.6	3290.1
	SCL	5.51	4.99	3.89	23.97	4.66	3.83	3.22	12.23
Phase7	SDNN	62.35	18.51	59.86	74.84	62.94	22.40	64.72	93.05
	rMSSD	41.86	23.86	38.17	106.44	43.75	27.1	38.97	125.59
	LF (ms^2^)	1761.95	1273.08	1580.55	4904.47	1818.2	1856.8	1336.7	7646.2
	HF (ms^2^)	1102.03	1543	591.42	7082.26	1040	1729.4	424.41	8063.5
	SCL	5.19	5.38	3.76	26.35	4.12	3.76	2.45	12.36

Table 2 shows the AIC and AIC weights of fitted mixed-models for each considered autonomic index. The mixed-model with fixed Phase predictor (M48; see Formulae A in S1 File) resulted the preferred model to fit the rMSSD, LF, HF and SCL (see Table 2).

**Table 2:** The AIC model comparison analysis of the mixed-effects models (M_n_).

SDNN			rMSSD			LF			HF			SCL		
M_n_	AIC	AIC_w_	M_n_	AIC	AIC_w_	M_n_	AIC	AIC_w_	M_n_	AIC	AIC_w_	M_n_	AIC	AIC_w_
M46	2740.9	23.10%	M48	2573.0	38.47%	M48	5270.7	42.69%	M48	5208.7	37.04%	M48	1026.6	35.63%
M48	2741.2	19.38%	M47	2574.7	16.44%	M47	5272.6	16.61%	M47	5210.6	14.30%	M47	1028.6	13.22%
M47	2742.7	9.24%	M42	2574.8	15.39%	M42	5272.7	16.06%	M42	5210.6	14.26%	M42	1028.6	13.12%
M41	2742.7	9.21%	M44	2576.6	6.15%	M44	5274.6	6.12%	M44	5212.6	5.35%	M39	1029.2	9.89%
M42	2742.8	8.98%	M32	2576.9	5.43%	M27	5275.7	3.49%	M22	5213.2	4.02%	M32	1030.1	6.36%
M36	2743.2	7.30%	M39	2577.4	4.22%	M17	5276.2	2.72%	M32	5213.2	3.95%	M34	1030.4	5.42%
M32	2744.5	3.77%	M22	2578.1	3.02%	M34	5276.3	2.64%	M39	5213.4	3.63%	M44	1030.6	4.89%
M44	2744.5	3.68%	M27	2578.7	2.25%	M24	5276.4	2.50%	M0	5214.0	2.63%	M27	1032.1	2.34%
M31	2744.7	3.47%	M34	2578.9	2.00%	M19	5277.7	1.29%				M29	1032.4	1.99%
M39	2745.0	2.92%	M17	2578.9	1.95%	M12	5277.7	1.28%				M22	1032.7	1.68%
M27	2746.4	1.43%	M24	2579.9	1.21%	M14	5278.2	1.00%				M24	1033.5	1.12%
M34	2746.5	1.39%	M29	2580.7	0.83%	M46	5278.2	0.99%				M17	1034.3	0.78%
M26	2746.6	1.32%	M12	2580.7	0.81%	M7	5279.4	0.55%				M7	1034.4	0.72%
M22	2747.3	0.91%	M19	2580.9	0.73%	M0	5279.5	0.53%				M4	1034.6	0.66%
M21	2747.5	0.85%	M7	2582.4	0.34%							M2	1034.6	0.65%
M17	2748.1	0.61%	M14	2582.6	0.31%							M19	1035.3	0.47%
M16	2748.3	0.56%	M9	2584.3	0.13%							M9	1035.6	0.40%
M29	2748.4	0.53%	M2	2584.4	0.13%							M12	1036.3	0.29%
M24	2749.3	0.34%	M46	2585.8	0.06%							M14	1037.2	0.18%
M12	2750.1	0.23%	M4	2586.3	0.05%							M46	1039.2	0.06%
M19	2750.1	0.23%	M41	2587.7	0.02%							M36	1039.8	0.05%
M11	2750.3	0.21%	M36	2588.5	0.02%							M31	1041.0	0.03%
M7	2752.1	0.08%	M31	2590.0	0.01%							M41	1041.2	0.02%
M14	2752.1	0.08%	M21	2591.0	0.00%							M26	1043.0	0.01%
M6	2752.3	0.08%	M26	2591.7	0.00%							M21	1044.2	0.01%
M2	2754.0	0.03%	M16	2592.0	0.00%							M1	1045.2	0.00%
M9	2754.1	0.03%	M11	2593.7	0.00%							M16	1045.9	0.00%
M1	2754.1	0.03%	M6	2595.4	0.00%							M6	1046.3	0.00%
M4	2756.0	0.01%	M1	2597.4	0.00%							M11	1047.9	0.00%
M0	2771.1	0.00%	M0	2599.0	0.00%							M0	1212.1	0.00%

Given 50 candidate mixed-effects models (M_n;_ see Formulae A in S1 File), the best fitting models are reported in terms of AIC and AIC_weight_. Considering SDNN index of HRV, the best fitting model was our modeled expectations (Code M46, Formula: ***SDNN***∼***Phase*** * ***Group*** + (**1|*Individual***)). Conversely, considering rMSSD, LF, HF indices of the HRV and SCL, M48 (Formula: ***Index***∼***Phase*** + (**1|*Individual***)) was the best fitting model.

**Fig 1:**
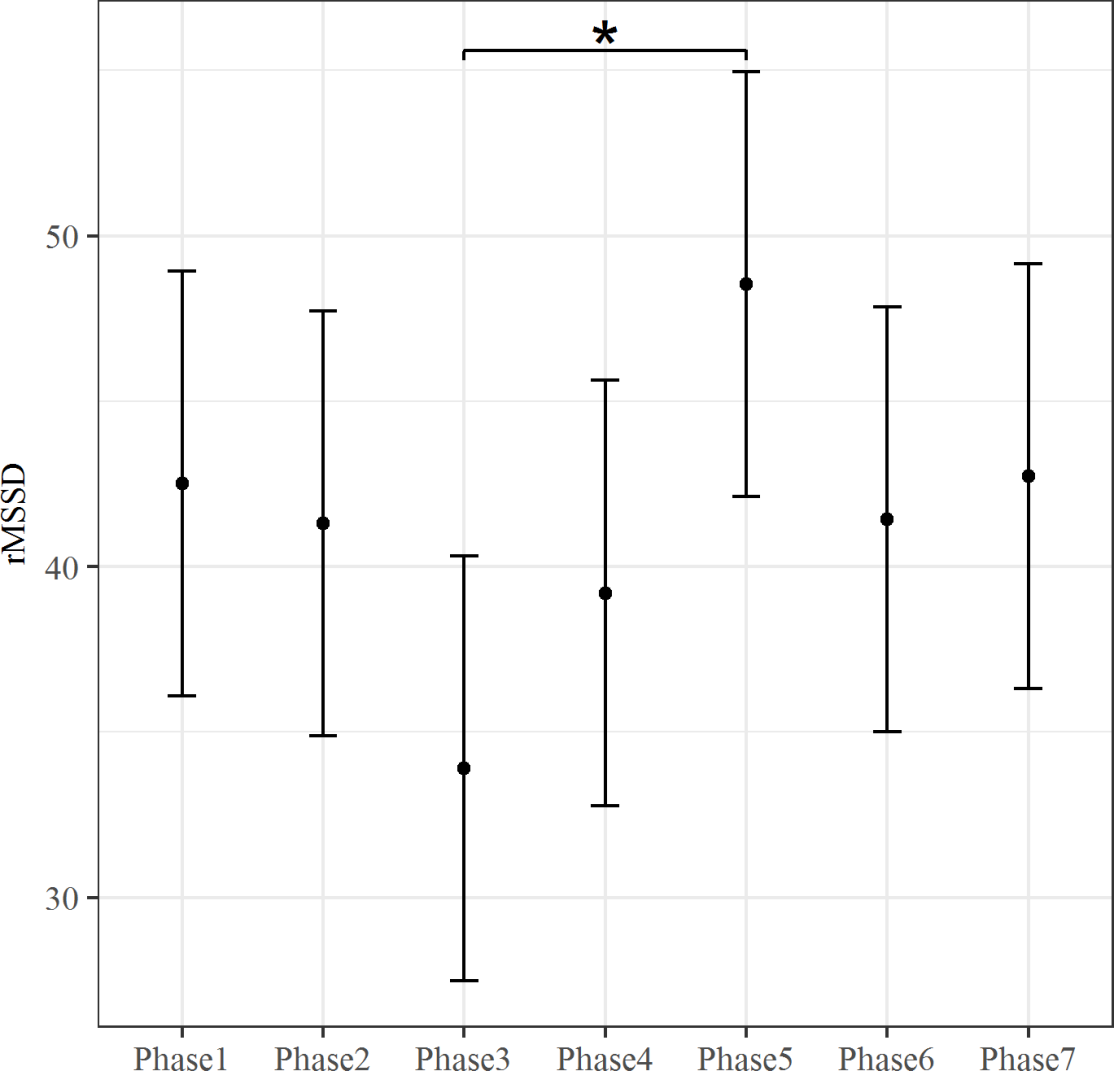
The effect of Phase on rMSSD. The bars at each data point represent the confidence limits computed at .95. Asterisks and lines indicate significant differences between Phase levels.

**Fig 2:**
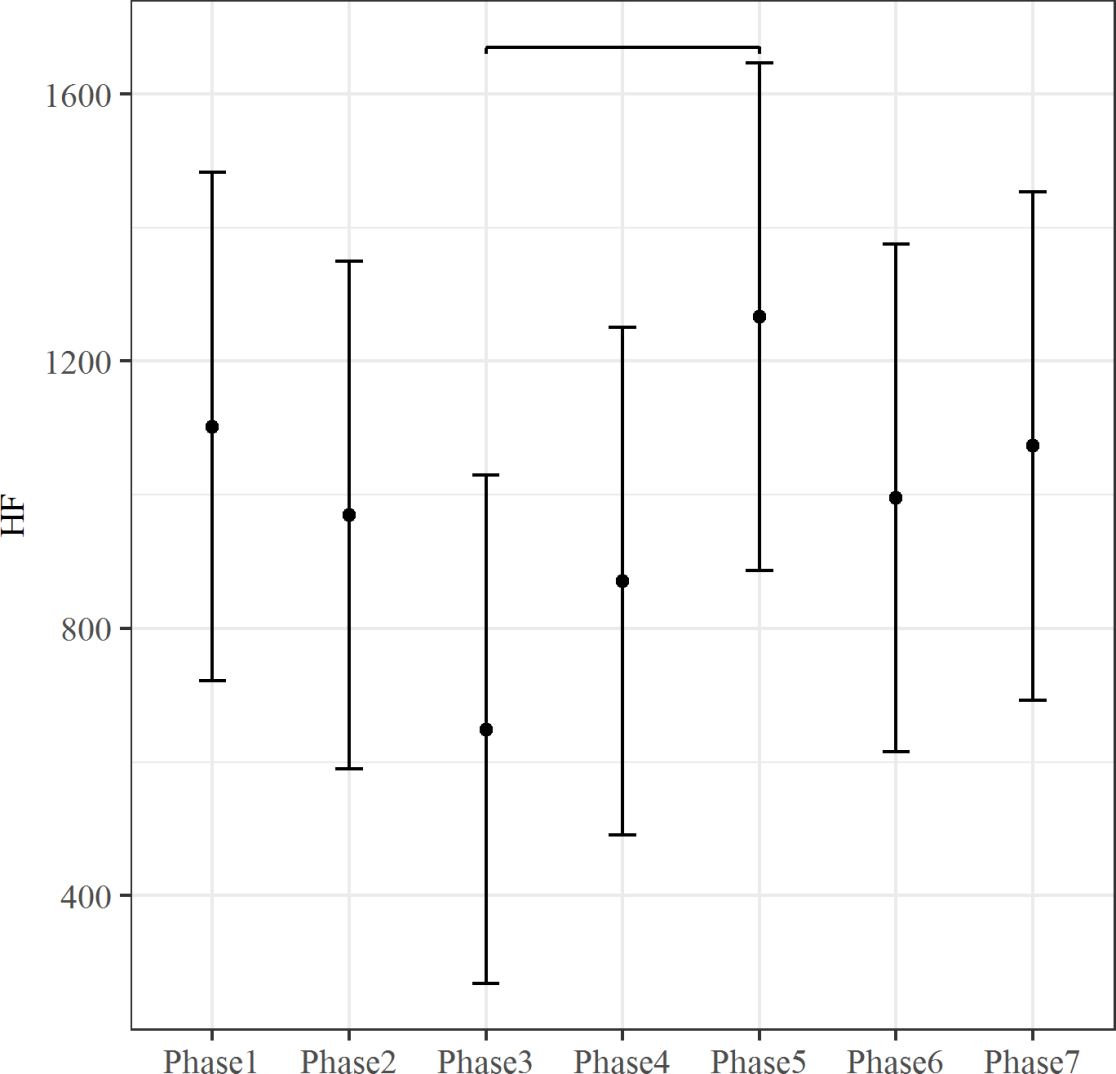
The effect of Phase on HF. The bars at each data point represent the confidence limits computed at .95. Asterisks and lines indicate significant differences between Phase levels.

**Fig 3:**
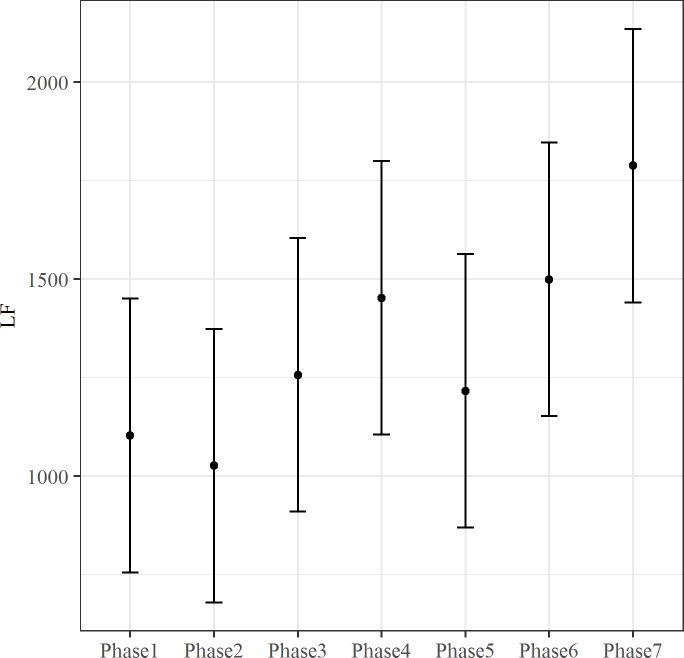
The effect of Phase on LF. The bars at each data point represent the confidence limits computed at .95. Asterisks and lines indicate significant differences between Phase levels.

**Fig 4:**
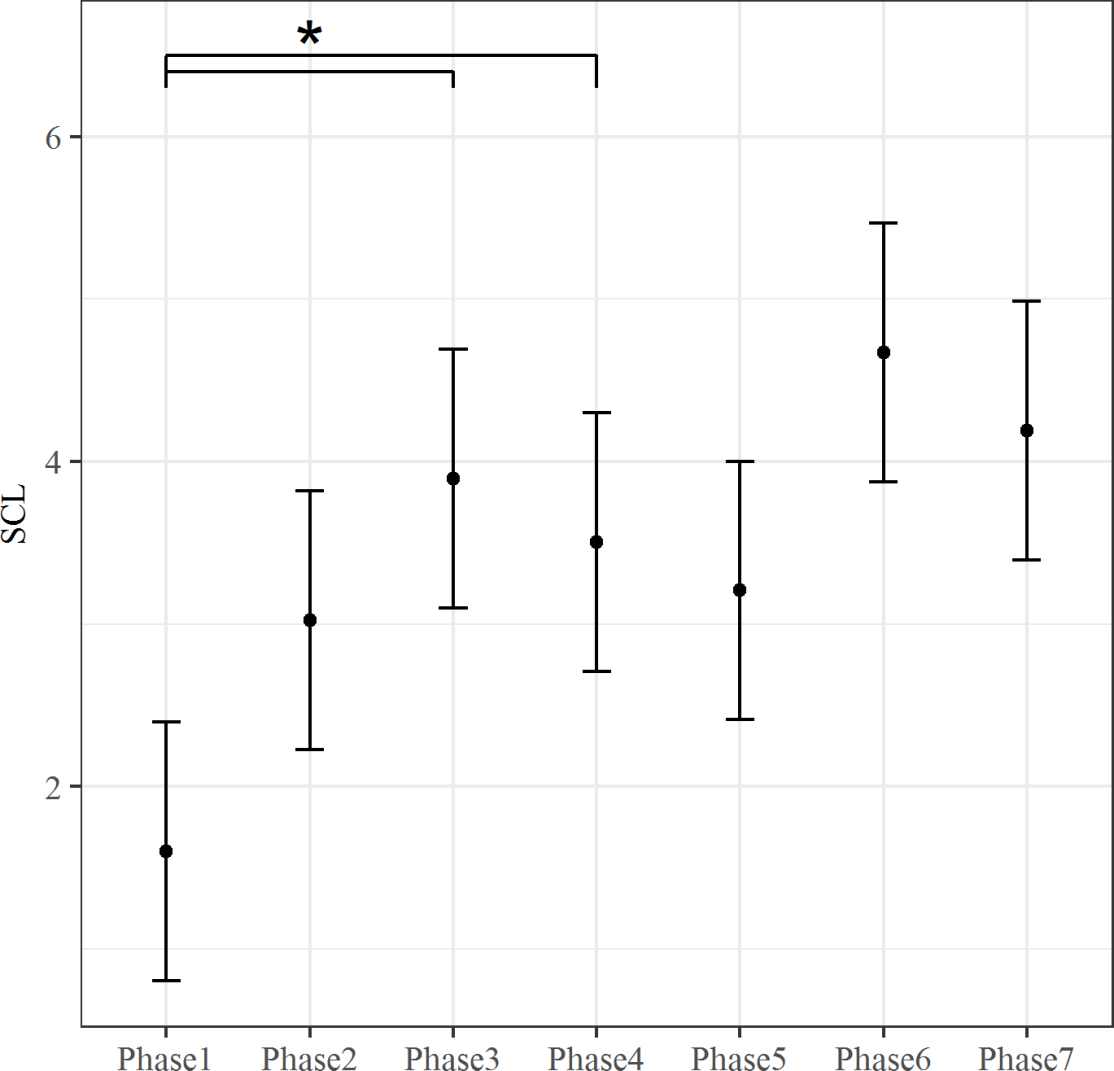
The effect of Phase on SCL. The bars at each data point represent the confidence limits computed at .95. Asterisks and lines indicate significant differences between Phase levels.

The effect of fixed predictor was tested by the maximum likelihood method. The inclusion of Phase predictor improved the fit of the model for rMSSD, LF, HF and SCL (rMSSD: ΔAIC = 26.03, *X*^*2*^ (6, N = 9) = 38.03, p < .001; LF: ΔAIC = 10.91, *X*^*2*^(6, N = 9) = 22.91 p < .01; HF: ΔAIC = 5.29, *X*^*2*^(6, N = 9) = 17.289 p < .01; SCL: ΔAIC = 185.51, *X*^*2*^(6, N = 9) = 197.51, p < .001). The Phase effect for these autonomic indices is showed in Figs 1–4. Both rMSSD and HF were lower during Phase 3 than Phase 5 (Figs 1 and 2, respectively). On the contrary, no significant differences between Phase levels were found for LF (Fig 3). Lastly, SCL was lower during Phase 1 than Phase 3 and Phase 4 (Fig 4).

**Fig 5:**
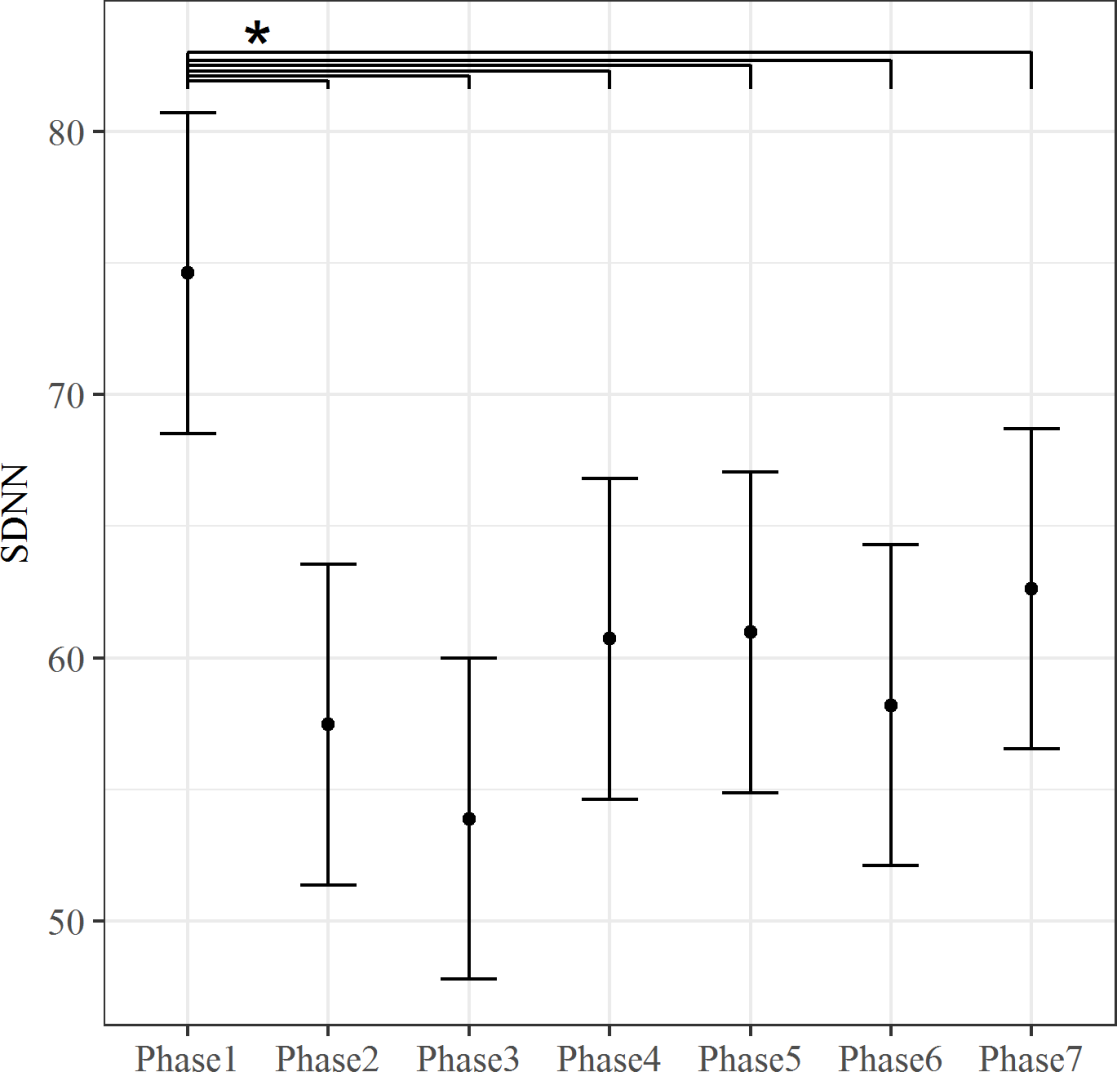
The effect of Phase on SDNN. The bars at each data point represent the confidence limits computed at .95. Asterisks and lines indicate significant differences between Groups for each level of the Phase predictor.

Different results were obtained considering SDNN. Our modeled expectations that considered Group, Phase and their interaction as fixed predictors (Model 46, see Formulae A in S1 File) resulted the best to describe the data. The inclusion of Phase predictor improved the fit of the model (ΔAIC = 29.91, *X*^*2*^(6, N = 10) = 41.91, p < .001), see Fig 5. SDNN was higher during Phase 1 than any other TSST phase.

**Fig 6:**
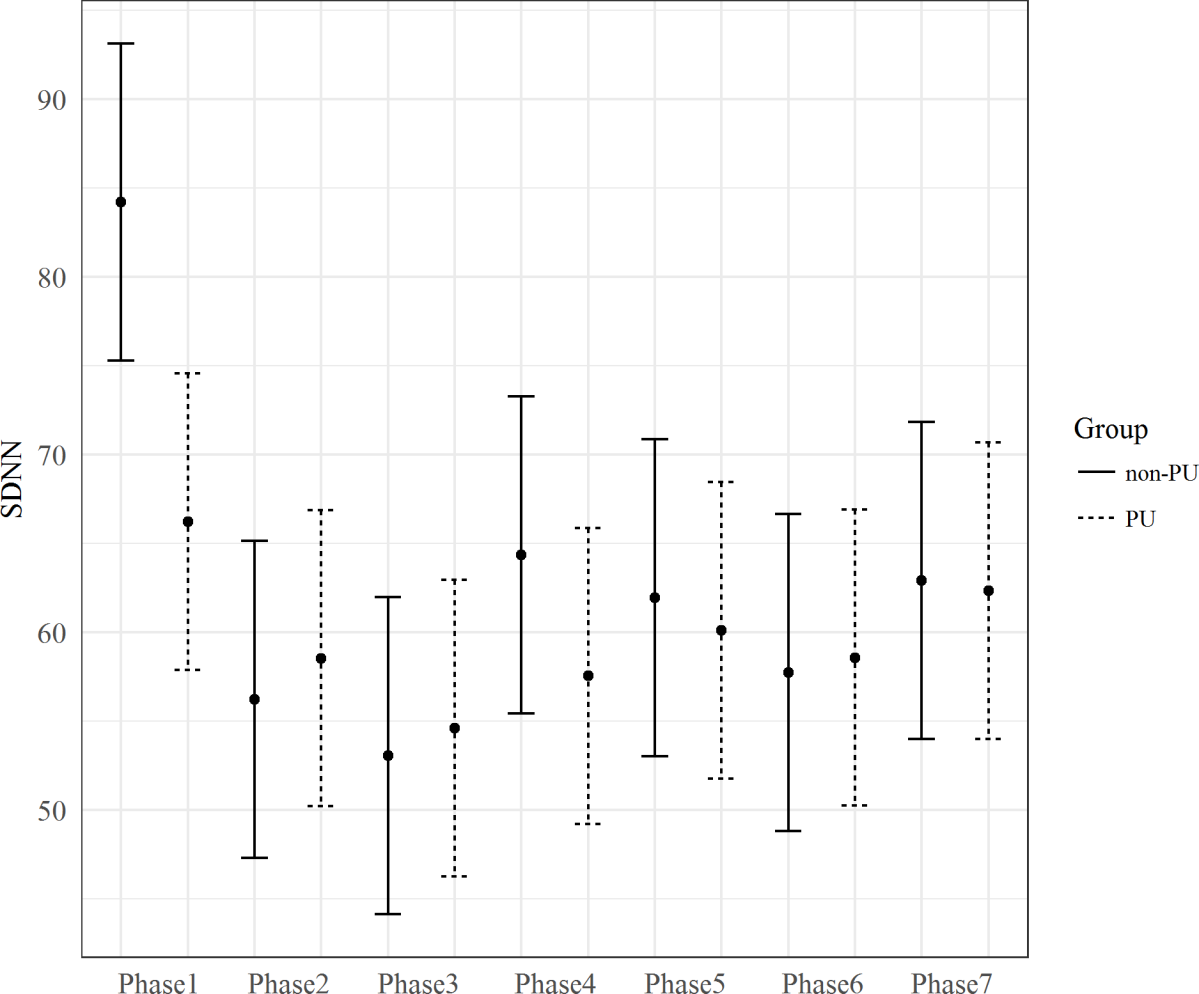
The Phase*Group interaction for SDNN. SDNN in each Phase and Group. The bars at each data point represent the confidence limits computed at .95. SDNN during Phase 1 was significantly greater in the non-PU than in the PU group. In the non-PU group, SDNN was greater during Phase 1 than in all the other Phases. No significant differences among Phases were found in the PU group.

No improvement in the fit of the model was found when Group was included as a predictor, however including the *Group* × *Phase* interaction resulted in an improvement in the fit of the model (ΔAIC = 1.83, *X*^*2*^(6, N = 16) = 13.83, p = .03), i.e., Groups and Phase predictors interact. As shown in Fig 6, during the first rest period (Phase 1) SDNN was lower in PU than non-PU. Moreover, SDNN during Phase 1 was higher than during any other TSST phase only among non-PU.

### Craving ratings

As indicated by the AIC selection method, the model without the *Group* × *Time* interaction term (L2; see Formulae B in S1 File) best fitted the data (Table 3).

**Table 3:** The AIC model comparison analysis of the ordinal logistic models (L_n_).

L_n_	AIC	AIC_w_
L2	191.5	47%
L4	192.2	34%
L1	193.5	18%
L3	206.2	0.3%
L0	206.4	0.3%

Based on the AIC and the AIC_weight_ of the ordinal logistic models (L_n_; see Formulae B in S1 File), L2 (Formula: *Craving*∼*Time* + *Group*) was the preferred model, indicating insufficient evidence to support a *Group* × *Time* interaction.

The ordinal logistic regression was significant only using Group to predict craving ratings: t = 3.89, p < .001, OR = 5.65, 95% CI = [0.88, 2.64]), indicating that PU were more likely to report higher craving ratings then non-PU. Time was found not to predict craving ratings (t = 1.62, p > .05, OR = 1.98, 95% CI = [-0.13, 1.52]).

**Fig 7:**
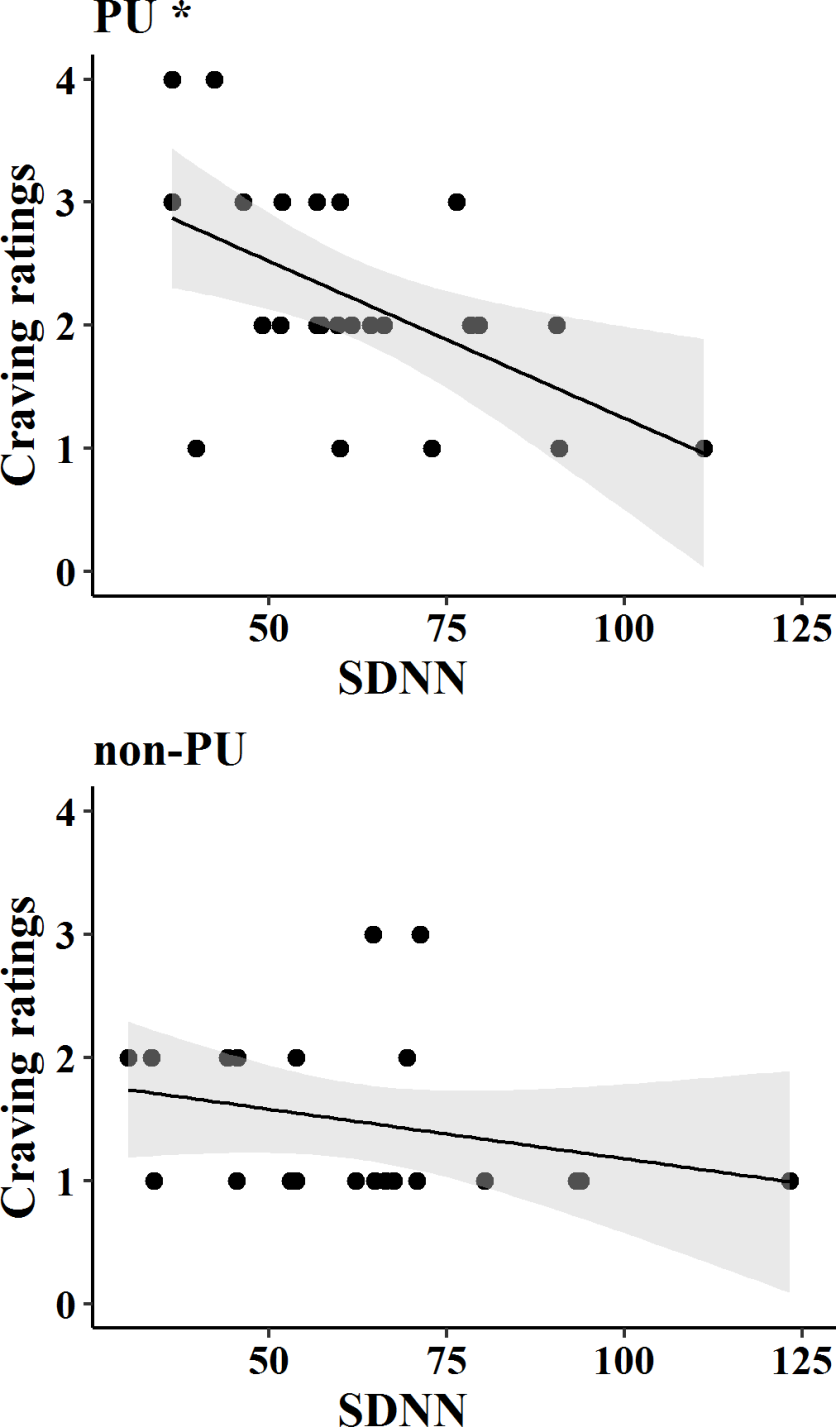
Correlation between craving ratings and SDNN after the stress task.

Finally, the Pearson correlation between SDNN measured during Phase 7 and craving ratings after the TSST showed a strong negative correlation between SDNN and craving ratings only among PU (r(24) = —.53, p < .01), see Fig 7.

### Self-report measures

Descriptive statistics of self-report measures are reported in Table 4.

**Table 4:** Descriptive statistics of self-report questionnaires.

	PU					Non-PU				
	n	mean	sd	median	range	n	mean	sd	median	range
AUDIT	24	8.08	4.26	8	20	21	4.48	4.24	3	19
CAST	24	0.75	2.25	0	11	21	0.67	1.6	0	7
DASS-21	24	21.88	11.67	17	39	21	12	6.83	12	24
BIS-11	24	61.75	9.43	63.5	31	21	54.95	6.5	56	23
NU	24	11.04	2.36	11	8	21	10.14	2.99	10	9
PU	24	10.04	2.37	10	11	21	9.1	2.59	8	9
LoPRE	24	8.63	3.46	8.5	12	21	6.33	1.83	6	5
LoPER	24	9.25	3.19	9.5	11	21	5.9	2.21	5	7
SS	24	10.21	2.96	10.5	11	21	8.86	2.87	9	11
OCI.R	24	16.67	7.04	16.5	27	21	8.24	3.67	8	17
TAS-20	24	47.67	10.37	46.5	45	21	38.1	8.94	35	35

**Fig 8:**
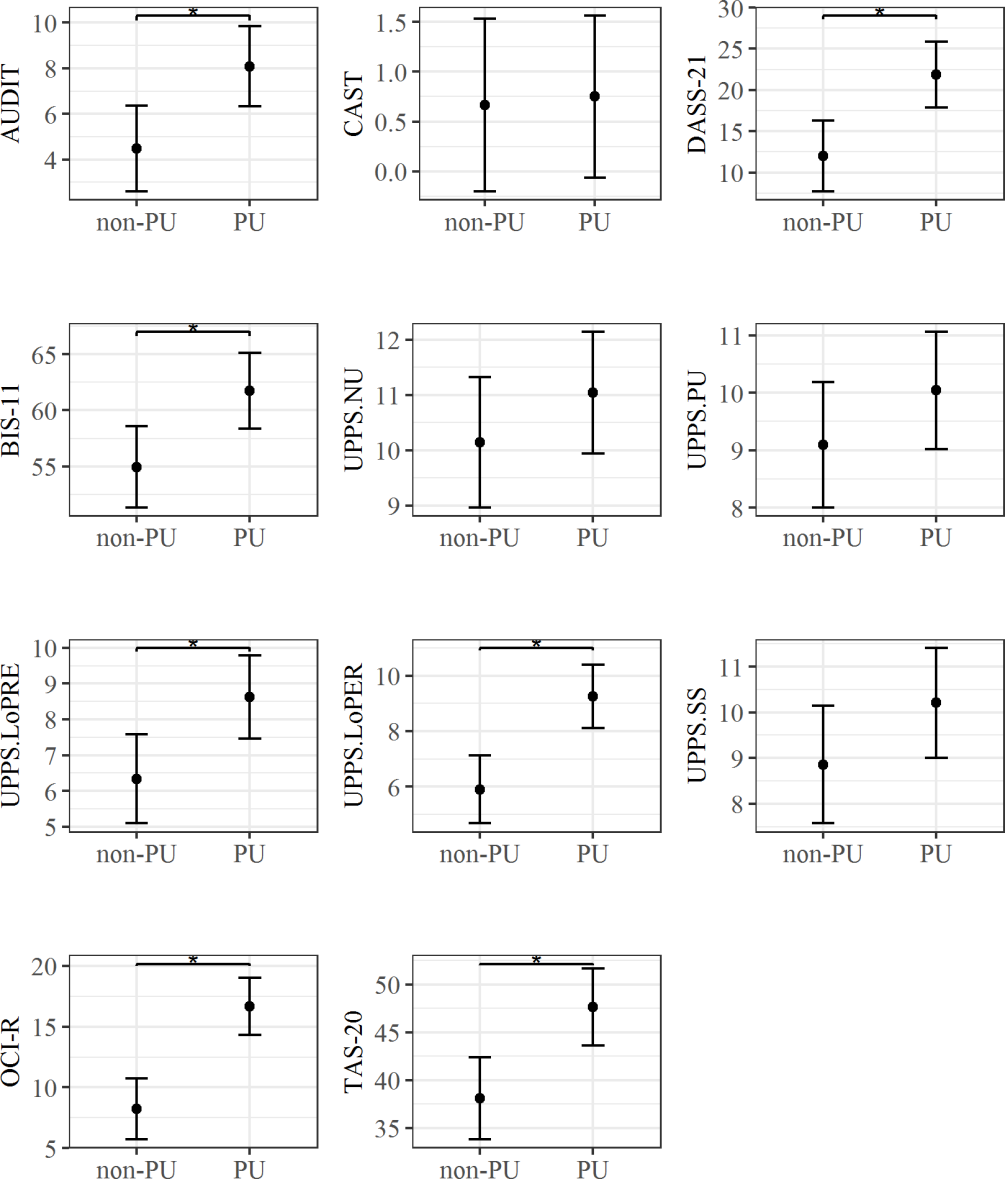
Significant group differences for self-report measures. The bars at each data point represent the confidence limits computed at .95. Asterisks and lines indicate significant differences between groups.

As shown in Fig 8, linear model analysis revealed higher scorings in the PU than the non-PU group in the AUDIT (F(1) = 8.06, p < .01, R^2^ = .16, BF = 6.57), the DASS-21 total score (F(1) = 11.54, p < .01, R^2^ = .21, BF = 22.62 ±0%), the BIS-11 (F(1) = 7.7, p < .01, R^2^ = .15, BF = 5.74 ±0%); the lack of premeditation (F(1) = 7.39, p < .01, R^2^ = .15, BF = 5.13 ±0%) and lack of perseverance (F(1) = 16.22, p < .001, R^2^ = .27, BF = 108.86 ±0%) components of the UPPS, the OCI-R (F(1) = 24.28, p < .001, Multiple R^2^ = .36, BF = 1308.96 ±0) and the TAS-20 (F(1) = 10.84, p< .01, R^2^ = .2, BF = 17.69 ±0%).

## Discussion and conclusions

This is the first study to our knowledge to investigate the relationship between autonomic stress reactivity and Internet craving in PIU. Specifically, we wanted to investigate (i) whether individuals with PIU show enhanced autonomic reactivity (i.e., lower HRV and higher SCL) to a standardized psychosocial stress task, (ii) whether greater autonomic reactivity is related to higher craving ratings, and (iii) whether PIU is associated with dysfunctional psychological features.

Contrary to our expectations, we did not find any group difference during the stress tasks. It may be hypothesized that the version of the TSST used in this study was not stressful enough to highlight possible differences in autonomic reactivity between individuals with vs without PIU. Moreover, the participants with PIU were recruited using the cut-off scores of the IAT, and were classified as occasional or frequent problematic Internet users. As such, they may not be fully representative of problematic Internet users. Future studies should include participants with severe problematic Internet usage to better elucidate autonomic stress reactivity in PIU.

We found that SDNN, that reflects the activity of all the cyclic components responsible for HRV [34], was lower in PU than non-PU before, but not during and after, the stress task. Lower HRV before the stress task suggests that, in PIU, reduced autonomic flexibility and impaired control of emotions may represent a stable condition, that is evidenced even in non-stressful conditions. Studies on SUDs suggest that regular and chronic use of drugs is associated with adaptations in stress-related brain pathways (specifically, the hypothalamic-pituitary-adrenal axis and autonomic nervous system pathways) [60]. It might be hypothesized that, similarly to substance addictions, behavioral addictions (including PIU) adversely impact autonomic functioning, reducing HRV at rest. On the other hand, low HRV in PU might be a vulnerability factor that underlies difficulty in self-regulation and inhibitory capacity [61], leading to problems in controlling one’s use of the Internet. Future research aimed at preventing and treating PIU should investigate whether low HRV represents a risk factor or a consequence of PIU.

The fact that we found group differences only for SDNN, reflecting both sympathetic and parasympathetic activity, but neither for other HRV indices nor for SCL, suggests that PIU is associated with an overall autonomic unbalance, rather than a specific dysregulation related to the sympathetic or the parasympathetic nervous system.

As regards the second research question, self-reported craving for Internet usage was higher in individuals with PIU than those without PIU, both before and after the stress task. Furthermore, after the stressful task, higher craving ratings were related to lower HRV only in PU. These findings support our hypothesis about the relationship between lower HRV and higher craving for Internet usage, suggesting that lower HRV in PU may be related to reduced capacity for self-regulation and ability to inhibit craving. Of note, these results fit with previous research showing that lower resting-state HRV predicted higher craving in alcohol dependent outpatients [62]. Overall, our findings generate new insight into the study of PIU by adding further support to the existence of a relationship between HRV and craving. However, the nature of the relationship between these variables is not currently understood. Future studies should further investigate the nature of this relationship in both behavioral and substance addictions.

Lastly, we found that PU endorsed more mood, obsessive and compulsive, and alcohol-related problems. Overall, these results are in line with previous findings showing that Internet addiction is associated with depression, anxiety, and stress [33,63], problematic alcohol use [64]; and obsessive-compulsive symptoms [16].

In addition to the above-mentioned limitations related to the task and to the criteria employed for sample selection, a further limitation of the current study is represented by the fact that we employed a single-item scale to collect Internet craving ratings [65,66]. Although this is considered as a sensitive method to measure craving, the combination with a questionnaire that explores the construct of craving through multiple items would improve the accuracy of the measure [67].

In conclusion, our findings provide new insights into the relationship between stress reactivity and craving in PIU, by supporting the existence of a relationship between reduced autonomic flexibility and Internet craving. Finally, our results confirm the previously reported associations of PIU with mood, obsessive-compulsive, and alcohol-related problems.

## Supporting information

S1 File **Formulae A. Mixed-effects models.** Legend: Index = HRV indices (i.e., SDNN; rMSSD; LF(ms^2^); HF(ms^2^)) and SCL; Phase = the Phase predictor (i.e. 1, 2, 3, 4, 5, 6 and 7 phases of the TSST); Group = the Group predictor (non-PU and PU); Individual = participants; AUDIT = the AUDIT ratings; DASS.D, DASS.A, and DASS.S = depression, anxiety, and stress subscales of the DASS-21 respectively; DASS.T = the DASS total score; LoPER and LoPRE = the lack of perseverance and lack of premeditation components of the UPPS-P, respectively; OCI = the OCI-R total score and TAS = the TAS score. **Formulae B. Ordinal logistic regression models.** Legend: Craving = Craving ratings; Time = Time predictor (i.e., before and after TSST); Group = Group Predictor (i.e., non-PU and PU).
(DOCX)

S2 File The recorded data. (ZIP)
