# Supplementary material for: Autonomic stress reactivity and craving in individuals with problematic Internet use
Source: PLoS One. 2018 Jan 16;13(1):e0190951. doi: 10.1371/journal.pone.0190951 (PMC5770068; doi:10.1371/journal.pone.0190951)
Supplement: S1 File — Formulae A. Mixed-effects models. Legend: Index = HRV indices (i.e., SDNN; rMSSD; LF(ms2); HF(ms2)) and SCL; Phase = the Phase predictor (i.e. 1, 2, 3, 4, 5, 6 and 7 phases of the TSST); Group = the Group predictor (non-PU and PU); Individual = participants; AUDIT = the AUDIT ratings; DASS.D, DASS.A, and DASS.S = depression, anxiety, and stress subscales of the DASS-21 respectively; DASS.T = the DASS total score; LoPER and LoPRE = the lack of perseverance and lack of premeditation components of the UPPS-P, respectively; OCI = the OCI-R total score and TAS = the TAS score. Formulae B. Ordinal logistic regression models. Legend: Craving = Craving ratings; Time = Time predictor (i.e., before and after TSST); Group = Group Predictor (i.e., non-PU and PU). (DOCX) [file pone.0190951.s001.docx]

**Supporting Information**

**Formulae A. Mixed-effects models (by lmer {lme4})**

M1 Model1: Formula = Index ~ Phase*Group + AUDIT + DASS.D + DASS.A + DASS.S + DASS.T + LoPRE + LoPER + OCI + TAS +(1|Individual)

M2 Model2: Formula = Index ~ Phase + AUDIT + DASS.D + DASS.A + DASS.S + DASS.T + LoPRE + LoPER + OCI + TAS +(1|Individual)

M3 Model3: Formula = Index ~ Group + AUDIT + DASS.D + DASS.A + DASS.S + DASS.T + LoPRE + LoPER + OCI + TAS +(1|Individual)

M4 Model4: Formula = Index ~ Phase + Group + AUDIT + DASS.D + DASS.A + DASS.S + DASS.T + LoPRE + LoPER + OCI + TAS +(1|Individual)

M5 Model5: Formula = Index ~ AUDIT + DASS.D + DASS.A + DASS.S + DASS.T + LoPRE + LoPER + OCI + TAS +(1|Individual)

M6 Model6: Formula = Index ~ Phase*Group + DASS.D + DASS.A + DASS.S + DASS.T + LoPRE + LoPER + OCI + TAS +(1|Individual)

M7 Model7: Formula = Index ~ Phase + DASS.D + DASS.A + DASS.S + DASS.T + LoPRE + LoPER + OCI + TAS +(1|Individual)

M8 Model8: Formula = Index ~ Group + DASS.D + DASS.A + DASS.S + DASS.T + LoPRE + LoPER + OCI + TAS +(1|Individual)

M9 Model9: Formula = Index ~ Phase + Group + DASS.D + DASS.A + DASS.S + DASS.T + LoPRE + LoPER + OCI + TAS +(1|Individual)

M10 Model10: Formula = Index ~ DASS.D + DASS.A + DASS.S + DASS.T + LoPRE + LoPER + OCI + TAS +(1|Individual)

M11 Model11: Formula = Index ~ Phase*Group + DASS.A + DASS.S + DASS.T + LoPRE + LoPER + OCI + TAS +(1|Individual)

M12 Model12: Formula = Index ~ Phase + DASS.A + DASS.S + DASS.T + LoPRE + LoPER + OCI + TAS +(1|Individual)

M13 Model13: Formula = Index ~ Group + DASS.A + DASS.S + DASS.T + LoPRE + LoPER + OCI + TAS +(1|Individual)

M14 Model14: Formula = Index ~ Phase + Group + DASS.A + DASS.S + DASS.T + LoPRE + LoPER + OCI + TAS +(1|Individual)

M15 Model15: Formula = Index ~ DASS.A + DASS.S + DASS.T + LoPRE + LoPER + OCI + TAS +(1|Individual)

M16 Model16: Formula = Index ~ Phase*Group + DASS.S + DASS.T + LoPRE + LoPER + OCI + TAS +(1|Individual)

M17 Model17: Formula = Index ~ Phase + DASS.S + DASS.T + LoPRE + LoPER + OCI + TAS +(1|Individual)

M18 Model18: Formula = Index ~ Group + DASS.S + DASS.T + LoPRE + LoPER + OCI + TAS +(1|Individual)

M19 Model19: Formula = Index ~ Phase + Group + DASS.S + DASS.T + LoPRE + LoPER + OCI + TAS +(1|Individual)

M20 Model20: Formula = Index ~ DASS.S + DASS.T + LoPRE + LoPER + OCI + TAS +(1|Individual), data=IAdata, REML=FALSE

M21 Model21: Formula = Index ~ Phase*Group + DASS.T + LoPRE + LoPER + OCI + TAS +(1|Individual)

M22 Model22: Formula = Index ~ Phase + DASS.T + LoPRE + LoPER + OCI + TAS +(1|Individual)

M23 Model23: Formula = Index ~ Group + DASS.T + LoPRE + LoPER + OCI + TAS +(1|Individual)

M24 Model24: Formula = Index ~ Phase + Group + DASS.T + LoPRE + LoPER + OCI + TAS +(1|Individual)

M25 Model25: Formula = Index ~ DASS.T + LoPRE + LoPER + OCI + TAS +(1|Individual)

M26 Model26: Formula = Index ~ Phase*Group + LoPRE + LoPER + OCI + TAS +(1|Individual)

M27 Model27: Formula = Index ~ Phase + LoPRE + LoPER + OCI + TAS +(1|Individual)

M28 Model28: Formula = Index ~ Group + LoPRE + LoPER + OCI + TAS +(1|Individual)

M29 Model29: Formula = Index ~ Phase + Group + LoPRE + LoPER + OCI + TAS +(1|Individual)

M30 Model30: Formula = Index ~ LoPRE + LoPER + OCI + TAS +(1|Individual)

M31 Model31: Formula = Index ~ Phase*Group + LoPER + OCI + TAS +(1|Individual)

M32 Model32: Formula = Index ~ Phase + LoPER + OCI + TAS +(1|Individual)

M33 Model33: Formula = Index ~ Group + LoPER + OCI + TAS +(1|Individual)

M34 Model34: Formula = Index ~ Phase + Group + LoPER + OCI + TAS +(1|Individual)

M35 Model35: Formula = Index ~ LoPER + OCI + TAS +(1|Individual)

M36 Model36: Formula = Index ~ Phase*Group + OCI + TAS +(1|Individual)

M37 Model37: Formula = Index ~ Phase + OCI + TAS +(1|Individual)

M38 Model38: Formula = Index ~ Group + OCI + TAS +(1|Individual)

M39 Model39: Formula = Index ~ Phase + Group + OCI + TAS +(1|Individual)

M40 Model40: Formula = Index ~ OCI + TAS +(1|Individual)

M41 Model41: Formula = Index ~ Phase*Group + TAS +(1|Individual)

M42 Model42: Formula = Index ~ Phase + TAS +(1|Individual)

M43 Model43: Formula = Index ~ Group + TAS +(1|Individual)

M44 Model44: Formula = Index ~ Phase + Group + TAS +(1|Individual)

M45 Model45: Formula = Index ~ TAS +(1|Individual)

M46 **Model46: Formula = Index ~ Phase*Group + (1|Individual)**

M47 Model47: Formula = Index ~ Phase + Group + (1|Individual)

M48 Model48: Formula = Index ~ Phase + (1|Individual)

M49 Model49: Formula = Index ~ Group + (1|Individual)

M0 Model0: Formula = Index ~ (1|Individual)

**Formulae B. Ordinal logistic regression models (by polr {MASS})**

L1 Model1: Formula = Craving ~ Time * Group

L2 Model2: Formula = Craving ~ Time + Group

L3 Model3: Formula = Craving ~ Time

L4 Model4: Formula = Craving ~ Group

L0 Model5: Formula = Craving ~ 1
